# Emotional face expression recognition in problematic Internet use and excessive smartphone use: task-based fMRI study

**DOI:** 10.1038/s41598-022-27172-0

**Published:** 2023-01-07

**Authors:** Ákos Arató, Szilvia Anett Nagy, Gábor Perlaki, Gergely Orsi, Anna Tímea Szente, Gréta Kis-Jakab, Eszter Áfra, Husamalddin Ali Alhour, Norbert Kovács, József Janszky, Gergely Darnai

**Affiliations:** 1 Department of Neurology, Medical School, University of Pécs, Rét Street 2, 7623 Pecs, Hungary; 2 ELKH-PTE Clinical Neuroscience MR Research Group, Pecs, Hungary; 3 Pécs Diagnostic Centre, Pecs, Hungary; 4 Department of Neurosurgery, Medical School, University of Pécs, Pecs, Hungary; 5 Neurobiology of Stress Research Group, Szentágothai Research Centre, University of Pécs, Pecs, Hungary; 6 Department of Laboratory Medicine, Medical School, University of Pécs, Pecs, Hungary; 7 Department of Behavioural Sciences, Medical School, University of Pécs, Pecs, Hungary

**Keywords:** Amygdala, Cognitive neuroscience, Emotion, Social neuroscience, Neuroscience, Psychology, Addiction

## Abstract

Growing literature indicates that problematic Internet use (PIU) and excessive smartphone use (ESU) are associated with breakdown of functional brain networks. The effects of PIU&ESU on emotional face expression (EFE) recognition are not well understood, however behavioural investigations and fMRI studies of different addiction forms indicated the impairment of this function. The Facial Emotion Recognition Paradigm was used to probe cortico-limbic responses during EFE recognition. Combined fMRI and psychophysiological analysis were implemented to measure EFE-related functional brain changes in PIU&ESU. Self-reported questionnaires were used to assess PIU&ESU. Positive associations were found between the extent of PIU&ESU and functional connections related to emotional cognitive control and social brain networks. Our findings highlight the involvement of social functioning, especially EFE recognition in PIU&ESU. Therefore, we emphasize that besides the brain’s executive and reward systems, the social brain network might be the next candidate to be involved in the pathogenesis of PIU&ESU.

## Introduction

Since Internet is an integrated part of our personal and professional lives, investigation of problematic Internet use (PIU) has proliferated in scientific community. PIU is generally defined as a behavioural addiction^1,2^, that shares characteristics with substance and other behavioural addiction forms. PIU includes uncontrolled Internet use, obsessive thinking about the Internet and neglecting every-day and social life^3^. PIU could lead to negative consequences in social interactions^4^, in daily life functions^5^, and also in professional performance^6^. Currently it is estimated that 6% of the world’s population is affected by PIU^7^, suggesting that PIU is considered as a new and relatively fast growing mental health concern.

To understand the neural basis of PIU and other more specific Internet-related addiction forms such as excessive smartphone use (ESU), increased number of magnetic resonance imaging (MRI) investigations have been performed. These studies found structural alterations in the brain reward system^8,9^ and gray matter alterations in areas involved in decision making processes^10^. Besides structural changes, system-level functional alterations have also been revealed related to inhibitory control network in subjects with PIU^11^.

In the conditions of PIU and ESU (hereafter PIU&ESU), social cognitive functions may also be altered^12^. Social cognition refers to specific cognitive processes which help to decode primer social signals such as emotional facial expressions (EFEs)^13^. Emotional expressions provide necessary information about mental states, therefore the accurate perception of facial expressions is indispensable for successful social functioning^14,15^. In functional cooperation with several cortical and subcortical structures^16–20^ human amygdala plays a crucial role in facial expression recognition^21^. Amygdala and prefrontal brain structures such as the frontal pole (FP), the dorso-lateral prefrontal cortex and the superior frontal gyrus (SFG) establish an important functional cooperation within the emotional cognitive control network in the regulation and recognition of emotion via frontal regions' cognitive control functions^16,22,23^. The alterations of these FCs are related to emotional disturbances and the pathogenesis of different addictive disorders^24,25^. Via their social cognitive functions, the anterior (ACG) and posterior cingulate gyrus (PCG)^17^, the middle temporal gyrus (MTG)^18^ and the supramarginal gyrus (SMG)^26^ are also involved in facial expression recognition through their functional cooperation with the amygdala. Reduced recognition of EFEs has a negative effect on social cognitive processes in several forms of substance addictions^27^**.** Subjects with alcohol use disorder^28^ show functional alterations in the fronto-limbic circuity^29^, cocaine abusers have reduced task-based amygdala connectivity with the ACG during facial emotion recognition^30^ while methamphetamine addicts tend to have social cognitive deficits, which is underlined by increased task related activity in the ACG^31^.

Since PIU and substance disorders share several similarities, it is essential to investigate social functioning, especially the facial emotion recognition in PIU&ESU. Social cognitive deficits are even more likely to occur in PIU, due to the extensive social life and communication in online space. Poorer emotion recognition performance was found among social network site (SNS) addicts relative to controls^32^, furthermore, emotion recognition accuracy in subjects with internet gaming disorder (IGD) is an independent predictor of the extent of the addiction^33^.

The psychological and neural alterations of EFE recognition is not well understood in PIU&ESU since only a small number of researchers are engaged in the topic^34^. Chun et al.^35^ found that subjects with ESU show extensive deactivation in emotion-processing brain regions during facial emotion processing, which may be induced by a failure on cognitive control. Besides this, Cheng and his co-workers explored modified resting-state functional connections between the amygdala and brain regions related to emotional functioning (ACG, precuneus, dorsolateral prefrontal cortex) in subjects with PIU^34^. Based on these neuroimaging observations, it is highly needed to investigate amygdala’s functional connectivity (FC) during EFE recognition in PIU&ESU. To investigate these EFE recognition related FCs, the Facial Emotion Recognition Paradigm (FERP)^36^ is the most frequently used paradigm.

Therefore, in this study our aim was to investigate the effects of PIU and ESU on task-induced functional connections in young adults using fMRI during the FERP. Psychophysiological interaction analysis (PPI) was implemented to get a comprehensive view about the neural background. Since previous studies used only resting-state fMRI approaches to investigate functional connectivity changes, our primary aim was to use a task- and voxel-based method (PPI) to get a comprehensive view about the possible effect of PIU&ESU. Our secondary aim was to increase the sample size since previous studies reported small-to-medium sample sizes (n < 30 in all studies).

According to the previous behavioural, resting-state and task-based fMRI studies, we assume that there are positive associations between bilateral amygdala-based functional connections involved in EFE recognition and the extent of PIU and ESU.

## Methods

### Participants

**Figure 1:**
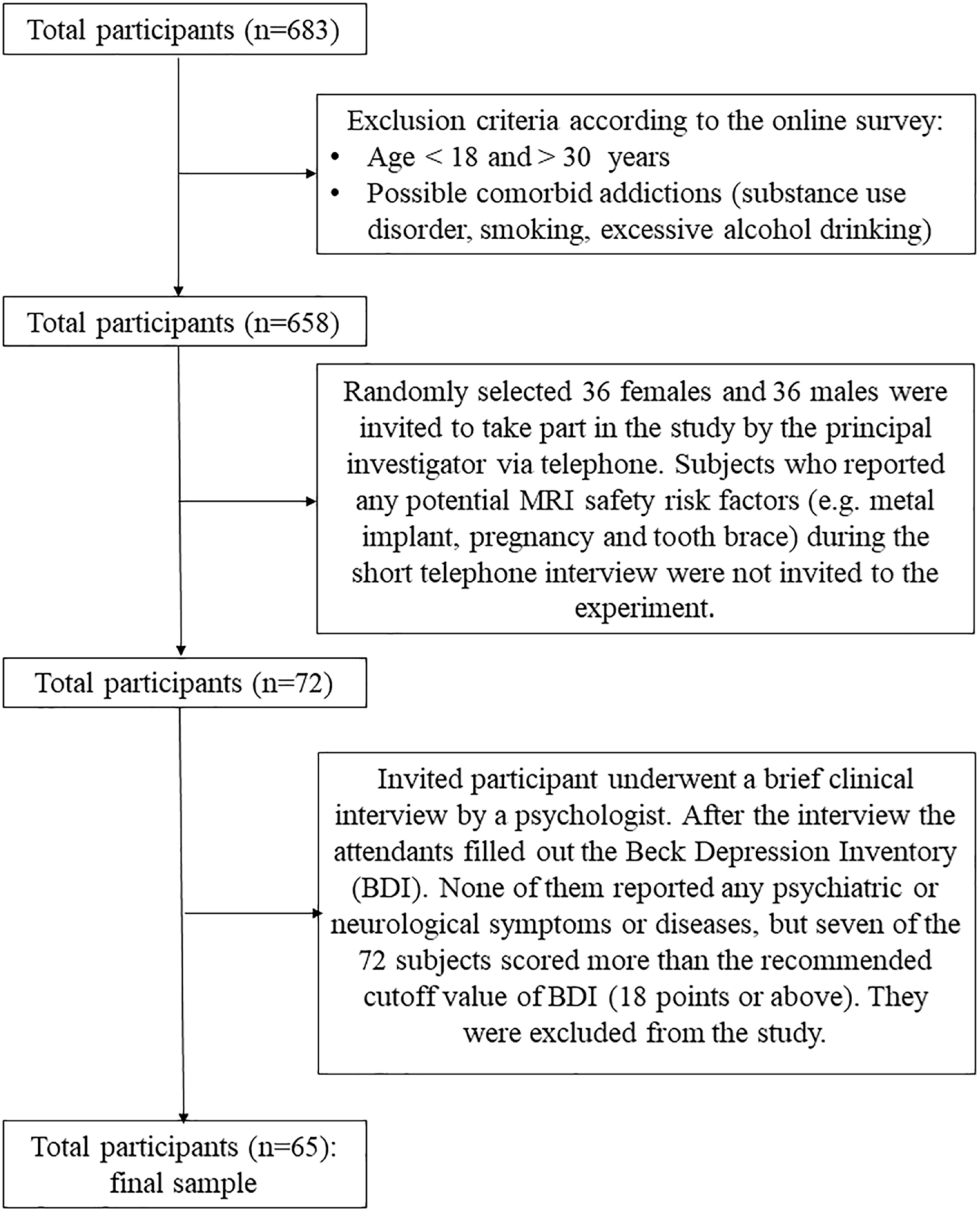
Selection of the final sample. A total of 683 participants completed the online survey, 25 of them reported smoking habits. They were excluded from the selection procedure. All candidates met the expected age range (18–30 years). From the remaining 658 participants 36 females and 36 males were chosen randomly with no MRI safety risk factors. Seven of the 72 subjects scored more than the recommended cutoff score of BDI, therefore they were excluded. Finally, 65 participants took part in the study.

All subjects were recruited through the Internet. A total of 683 adults participated in the online survey on problematic Internet use. After the inclusion procedure 65 Caucasian university students (32 males, mean age = 23.5, SD = 2.91; 33 females, mean age = 21.5, SD = 2), aged between 18 and 30 (mean age = 22.5, SD = 2.68) years were randomly chosen to take part in the study. During the selection process, subjects who reported any potential MRI safety risk factors, psychiatric or neurological symptoms were excluded. The handedness was assessed with the Edinburgh Handedness Inventory^37^. According to the scores, all participants had right-hand dominance (median, min–max; 80, 30–100). Depression was measured with the Beck Depression Inventory (BDI) and only participants scoring less than eighteen were included in the study^38^. According to self-reported questions subjects spend on average 3.38 (SD = 1.75) hours online per day. For a more detailed description of the selection process see Fig. 1.

Subjects were paid a small fee for their participation in the study. This research was approved by the Regional Research Ethics Committee (7476—PTE 2018). All procedures performed in studies involving human participants were in accordance with the ethical standards of the institutional and national research committee and with the 1964 Helsinki declaration and its later amendments. All subjects signed a written informed consent in the study.

## Assessment

### Problematic internet use questionnaire (PIUQ)

Without well-established diagnostic criteria, it is highly recommended to measure PIU with a multidimensional questionnaire without cutoff points^39^. Therefore, we did not form control and problematic user groups based on the severity of PIU, rather used it as a continuous variable.

The extent of PIU was measured with the Hungarian version of the PIUQ^3,40^. PIUQ is a self-reported inventory with good validity and reliability characteristics. The questionnaire contains 18 items, for each item scored on a 5-point Likert scale ranging from 1 (never) to 5 (always). PIUQ consists of three subscales which are obsession, neglect, and control disorder. Each subscale contains six items. The obsession scale measures the extent of the obsessive thinking of the Internet (e.g. fantasizing and daydreaming) and the withdrawal symptoms induced by the lack of the online state (e.g. depression, anxiety). Neglect refers to negation of daily activities and social life caused by excessive Internet use (e.g., reduced sleep time & essential needs intake). Control disorder subscale assesses the disability of controlling time spent on the Internet. The total score of PIUQ was computed by summing the scores of all three subscales. The overall internal reliability of the PIUQ is good (Cronbach alpha is 0.87)^3^.

### Smartphone application-based addiction scale (SABAS)

We used the Hungarian version of the SABAS to measure excessive smartphone use^41^. SABAS is a unidimensional self-reported inventory, with good internal consistency (Cronbach alpha = 0.82) and validity, comprises six items^41^. These items refer to the six core criteria of the addiction (salience, mood, modification, tolerance, withdrawal conflict and relapse)^42^. All the items are rated using a 6-point Likert-type scale ranging from 1 (strongly disagree) to 6 (strongly agree). The total score of SABAS was computed by summing all six items. Higher score in SABAS indicates a greater risk of developing addiction to smartphone use.

## Stimuli

### FACES database

Face photographs used in the FERP were selected from the FACES database^43^ which is a validated set of faces displaying different emotional states. Photographs depicted young adults (mean age = 24.2, SD = 3.4, age range = 19–31 years) on bright background, all wearing identical standard grey T-shirts without jewellery, glasses, make-up, or other eye-catching items. During their research Ebner and her colleagues found that the age of the face plays a crucial role in facial expression identification e.g., younger people identify young people’s facial expressions more precisely than older and middle-aged^43^. Therefore, we used images depicted young adults from the database due to our participants age range.

We used presentation software (Neurobehavioral System, Inc., Berkley, CA, USA) to run the paradigm during the fMRI measurement. The visual stimuli were presented on an MRI-compatible display system (Cambridge Research Systems Ltd, BoldScreen 24", Rochester, UK) by means of a mirror attached to the head coil. All responses from the subject were collected via MR-compatible response buttons (ResponseGrip, NordicNeuroLab AS, Bergen, Norway).

### Experimental design and paradigm

The Facial Emotion Recognition Paradigm (FERP) developed by Hariri and colleagues^36^ is a well-validated and effective task to probe corticolimbic responses during the identification of emotional facial expressions. According to previous studies the original FERP and its variants have been shown to reliably and robustly engage the amygdala and strongly activates corticolimbic networks involved in emotion generation and regulation^36,44,45^.

The FERP comprises two conditions. During the matching emotion condition, a target face (on the top of the display) and two test faces (bottom left and right) were presented in a triangular arrangement. Participants were instructed to choose one of the two test faces on the bottom that expressed the same emotion (anger, fear, sadness) as the target face on the top of the screen. All three faces were different, and an equal number of male and female faces were presented through the tasks. The target face and the congruent test face always displayed the same facial expression, the incongruent test face always showed neutral expression. Subjects made responses by pressing MR-compatible response buttons in either their left or right thumb in accordance with the position of the chosen probe face. Participants’ decisions were marked by a yellow square on the screen and once it was appeared, subjects were unable to change their response. As mentioned earlier, face photographs were selected from the FACES database^43^.

Blocks of matching face tasks were interspersed with blocks of a baseline task of matching geometric shapes (matching shape task). During the task participants completed trials involving emotional neutral, abstract geometric forms (circles, vertical and horizontal ellipses) in an analogous configuration like in matching emotion task. Subjects were asked to indicate which shape at the bottom matches the target form at the top. These trials served to maintain attention and allow corticolimbic brain responses to return to the baseline. Similarly, to the previous condition participants response by pressing MR-compatible response buttons in accordance with the position of the chosen test shape. A yellow square on the screen marked the participant’s unchangeable decision.

During the blocks participants’ responses and reaction times (RTs) were recorded.

The whole run consisted of six matching emotion and six matching shape alternating blocks. Each block lasted 30 s, containing six sequential matching trials. Each picture was presented for 5 s, with no interstimulus interval. The whole experiment with a try-out session lasted 370 s. Half of the trials required left-handed, while the other half required right-handed responses within each block.

**Figure 2:**
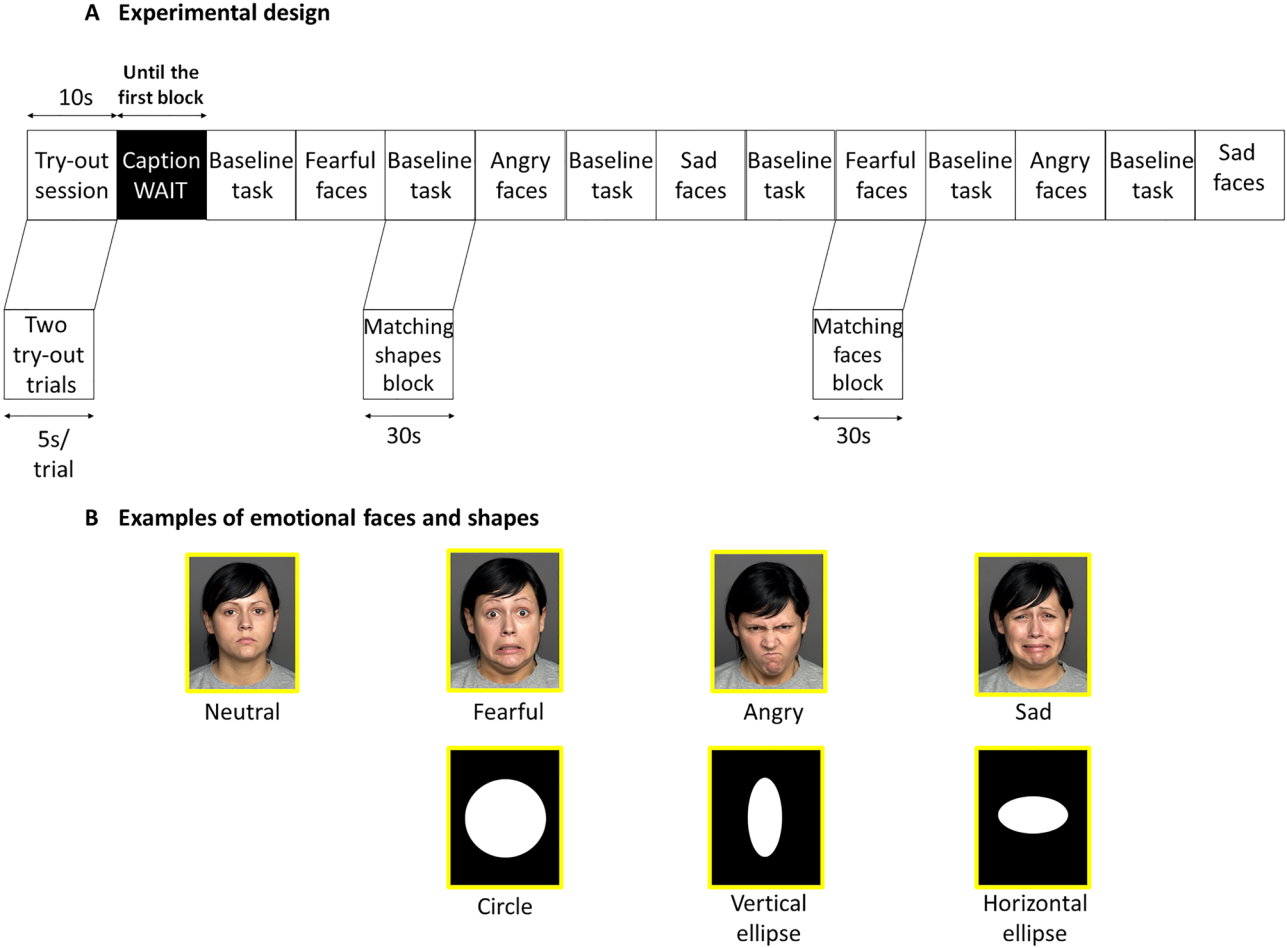
Schematic design of the Facial Emotion Recognition Paradigm^36^ and examples of emotional faces from FACES database^43^ and shapes. (**A**)**:** The figure shows that the experiment started with a try-out session in which participants solved one matching face and one matching shapes try-out trial. This was followed by task sessions (12 blocks) that were presented successively (first a baseline block which was always followed by a matching emotion block). Each 30 s block contained six matching trials and subjects were instructed to choose one of the two test faces presented at the bottom (left or right) that was identical to the target image (centre top position). Within each trial, the corresponding stimuli sets stayed on the screen for the entire 5 s, even after the participant made a choice. In all emotion block, the same target emotional expression was presented for six times across the trials. (**B**)**:** Examples of presented emotional faces with four different expressions (neutral, fearful, angry and sad) and geometric shapes (circle, vertical and horizontal ellipses).

The experiment started with an instruction period, followed by a try-out session which contained one trial of both conditions (matching emotion and matching shape). After the participant solved the try-out session caption “WAIT” appeared on the screen until the first matching shape block started. Within each trial, the corresponding stimuli sets stayed on the screen for the entire 5 s, even after the participant made a choice. The first matching shape block was followed by the first matching emotion block. Within each matching emotion block, the same target emotional expression was presented for six times. Half of the tasks were presented with male facial expressions, while the other half with female expressions in random order. During the six matching emotions blocks all three target facial expressions were presented twice in alternated order (for more details about the FERP and the experimental design, see Fig. 2).

### Imaging data acquisition and visual analysis

All measurements were performed on the same 3 T MRI scanner (MAGNETOM Prismafit Siemens Healthcare, Erlangen, Germany) with a 20-chanel Head/Neck coil.

Functional images were acquired using a 2D single-shot echo-planar imaging (EPI) sequence (TR/TE = 2500/30 ms; Flip angle = 76°; 36 axial slices with a thickness of 3 mm; FOV = 192 × 192 mm^2^; matrix size = 64 × 64; receiver bandwidth = 2170 Hz/ pixel; no gap, interleaved slice order to avoid crosstalk between contiguous slices). For distortion correction purposes, field mapping sequence (TR/TE1/TE2 = 400/4.92/7.38 ms; Flip angle = 60°; 36 axial slices; FOV = 210 × 210 mm^2^; matrix size = 70 × 70; receiver bandwidth = 290 Hz/pixel) with the same voxel size with 25% gap, orientation and adjustment parameters as the fMRI scan was acquired right after the fMRI measurement.

Anatomical images were obtained using an isotropic T1-weighted 3D-MPRAGE sequence (TR/TI/TE: 2530/1100/3.37 ms; Flip angle = 7°; 176 sagittal slices with thickness of 1 mm; FOV = 256 × 256 mm^2^; matrix size = 256 × 256; receiver bandwidth = 200 Hz/pixel). The MPRAGE anatomical images were visually checked by MRI experts. There were no brain abnormalities according to the visual inspection of the MR images.

### Functional MRI data pre-processing

The same preprocessing steps were used in BOLD signal analyses and FC analyses.

Pre-processing steps, BOLD signal and FC analyses were performed using freely available software tools within FMRIB’s Software Library FEAT Version 6.00, part of FSL (FMRIB’s Software Library, FSL, www.fmrib.ox.ac.uk/fsl).

Pre-processing procedure included MCFLIRT motion correction, brain extraction^46^, spatial smoothing (Gaussian kernel, 5 mm full width at half maximum), EPI distortion correction with FSL FUGUE^47^ and high-pass temporal filtering with 100 s cutoff.

Single-session data sets were registered into the MNI152 standard space using a two-step process. First, the functional image of each subject was registered to that subject’s high-resolution T1 structural scan using BBR (6 degrees-of-freedom)^48^. Then, each subject’s T1 image was registered to the 2 mm MNI152 standard space using 12 DOF linear fit followed by nonlinear registration (FNIRT, warp resolution = 10 mm)^49^. Next, for each subject, these two registrations were combined and applied to the first-level statistical maps to take them into standard space.

### BOLD signal analysis

BOLD analysis was performed to get a comprehensive view of the activation pattern during FERP’s matching emotion condition and extract bilateral amygdala seeds’ timeseries for PPI analysis. The whole brain general linear model (GLM) time-series statistical analysis of individual data sets was performed using FILM (FMRIB’s Improved Linear Model part of FSL).

First level analyses included a single regressor: modelling matching emotion conditions as task active periods and matching shape conditions as baseline periods.

Higher-level mixed-effect analyses were carried out using FLAME1 (FMRIB’s Local Analysis of Mixed Effects) with outlier de-weighting to investigate activation pattern during matching emotion condition.

**Figure 3:**
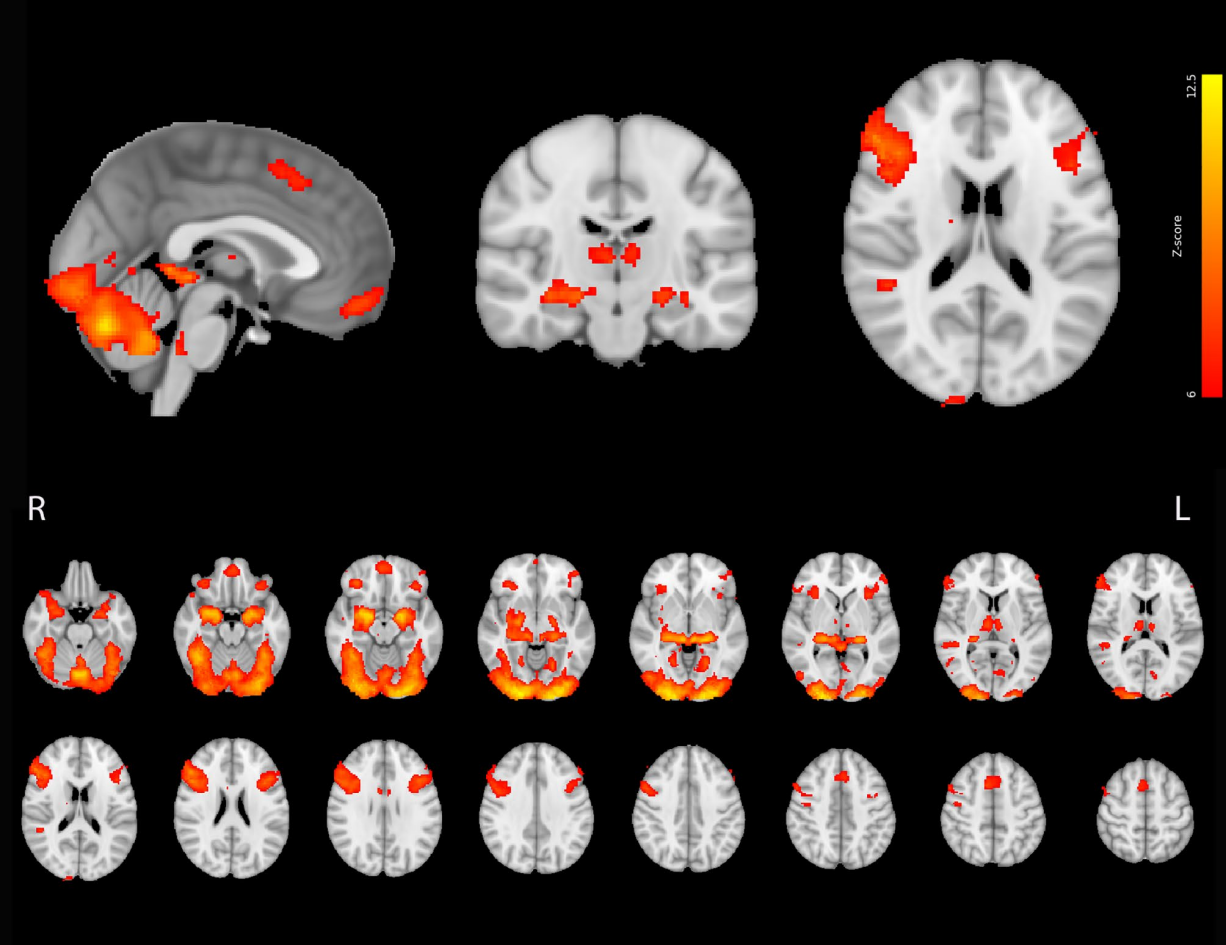
Mean task-dependent activations during the FERP. Image was thresholded using clusters determined by Z > 6 and a corrected cluster significance threshold of *p* = 0.05. Red–yellow colours indicate brain activation Z-scores during matching emotion tasks. Images are shown in radiological convention.

In order, to make interpretate our findings, mean activation map was calculated (Fig. 3). Statistical map was considered to be significant at Z > 6 and a family-wise error corrected cluster significance threshold of *p* = 0.05^50^. According to the activation map, similarly to previous studies^36,44^, the FERP successfully and robustly engaged the bilateral amygdala and strongly activated brain areas which are involved in emotional face expression recognition. According to the statistical map, we could certainly extract the amygdala seeds’ time-courses during match emotion condition in functional connectivity analysis.

### Functional connectivity analysis

To examine task related FC, we performed a whole brain PPI analysis in FSL using the pre-processing steps described above.

During the analyses we used left and right amygdala as seed regions.

PPI analysis is a method to finding correlation in neuronal activation between two distant brain structures in a given psychological context (i.e., in matching emotion task). In other words, PPI aims to capture an interaction between the psychological state (the behavioural task) and the functional coupling between two brain areas. In PPI, we principally look for regions which have a higher correlation with the time-course in the seed region in one psychological state (matching emotion tasks) than another (baseline, matching shape tasks). A task-specific increase in the correlation between a seed region and other voxels in the rest of the brain is suggestive of a task-dependent growth in the exchange of information^51^.

Our seed regions were structurally and functionally constrained.

1. At first step subcortical and cortical reconstruction and segmentation were implemented on subjects’ T1-weighted anatomical images with usage of Freesurfer 6.0 (https://surfer.nmr.mgh.harvard.edu/fswiki)^52^. After this procedure, visual quality control was performed by MRI experts. If error was found during reconstruction, error correction was implemented according to the recommended workflow (http://surfer.nmr.mgh.harvard.edu/fswiki/RecommendedReconstruction). Anatomical labels of amygdala (both left and right) were extracted for each participant separately based on Freesurfer’s subcortical segmentation atlas^53^.
2. Then these T1-weighted seed regions were transformed onto the participant’s functional EPI image through BBR linear registration with 6 DOF using FSL FLIRT (part of the FSL’ s toolbox).
3. To make sure that our seed regions were significantly active and engaged during the matching emotion task we excluded those voxels which did not show significant activations during the matching emotion task (Z < 2.3).
4. Finally, we extracted the average time-courses within our subject-specific left and right amygdala seed regions.

After the extraction of subject specific time-courses, PPI analyses were conducted for left and right amygdala seeds separately to define voxels in which activity is more correlated with the time-course in the seed regions in psychological context (i.e., in matching emotion condition), than another. For implementing PPI, during the standard GLM first-level analysis, we used instructions of FSL experts (https://fsl.fmrib.ox.ac.uk/fsl/fslwiki/PPIHowToRun). The design of first-level analysis contained three regressors. The first was the psychological regressor convolved with an HRF which represents task condition and is used to determine condition-specific changes in functional connections between regions. Second was the physiological regressor which represent time-courses taken from seed areas (left and right amygdala). The third variable was the interaction between previous regressors, which identified those voxels that showed greater correlation with the seed region’s time-course in psychological context than in the baseline condition. Time-series statistical analysis was carried out using FILM. In order to explore PIU related functional connections, region of interest (ROI) analyses was performed. During the analyses, FCs between the left and right amygdala and brain regions which are related to EFE recognition were investigated. For the analysis we defined ROIs a priory and extracted the parameter estimation (PE) values of all voxels within the ROIs from each participant’s subject-level PPI contrast, separately for left and right amygdalar seeds. All ROIs were defined according to previous studies, all of which showed to be involved in social cognitive and executive control processes during EFE recognition. These ROIs covered the following areas: *left and right superior frontal gyrus* (SFG)^22^, *left and right frontal pole* (FP)^54^, *left and right posterior and anterior cingulate gyrus* (ACG)*, left and right supramarginal gyrus* (SMG)^17^, *left and right middle temporal gyrus* (MTG)^18^. ROIs were defined based on the lateralized Harvard–Oxford Cortical Structural Atlas. Featquery was used to apply ROIs separately to each participant subject-level PPI contrast’s cope image. During analyses, left and right amygdala’s FCs’ PE values were extracted.

### Statistical analysis for behavioural and questionnaire data

Statistical analyses were performed using IBM SPSS 25.0 (IBM Corp. Released 2017. IBM SPSS Statistics for Windows, Version 25.0. Armonk, NY: IBM Corp).

Mean reaction times (RTs) with standard deviations (SD) and medians of the number of errors (ERs) with minimum and maximum values were calculated for each stimulus type in both matching emotion and matching shape conditions. Since none of our questionnaire data showed normal distribution, Spearman’s rank correlation with Benjamini–Hochberg correction (q = 0.05) was used to investigate the associations between the PIUQ total and subscales and the scores of SABAS.

Since none of the FC values showed normal distribution, Spearman’s rank correlations were applied to investigate the associations between PPI and questionnaire scores. To avoid false positives caused by multiple comparisons, Benjamini–Hochberg corrections (q = 0.05) were applied.

## Results

### Behavioural results

The participants’ demographic and questionnaire data are reported in Table 1.

**Table 1:** Descriptives of subjects’ demographic and questionnaire data.

Total number of subjects	All subjects	Males	Females
	N = 65	N = 32	N = 33
*Age (years)*			
Mean	22.50	23.50	21.50
SD	2.68	2.91	2.00
Educational level			
High school diploma	46	20	26
Bachelor’s degree	15	9	6
Master’s degree	4	3	1

	All subjects	Males	Females
*Questionnaire descriptives (median, min–max)*			
PIUQ total	31 (19–61)	32.5 (23–61)	29 (19–51)
PIUQ obsession	9 (6–22)	9 (6–22)	9 (6–17)
PIUQ neglect	11 (6–23)	11.5 (7–23)	10 (6–20)
PIUQ control disorder	11 (6–20)	11 (6–18)	10 (6–20)
SABAS	9 (6–24)	8.5 (6–20)	9 (6–24)

*N* number of participants; *PIUQ* Problematic Internet Use Questionnaire; *SABAS* Smartphone Application-Based Addiction Scale. Due to non-normal distribution, medians and min–max ranges are presented.

Valid behavioural data were available from all participants. According to the mean RTs and ERs subjects performed the FERP, thus no one was excluded from further analyses based on behavioural outcomes (Table 2). Moreover, no correlation was found between PIUQ scores and behavioural data (RTs and ERs).

**Table 2:** Behavioural outcomes of the FERP.

	Horizontal ellipse	Vertical ellipse	Circle	Overall shapes	Angry faces	Fearful faces	Sad faces	Overall faces
Reaction times (Mean ± SD)	0.763 ± 0.144	0.768 ± 0.160	0.736 ± 0.136	0.757 ± 0.138	1.066 ± 0.230	1.107 ± 0.230	1.252 ± 0.297	1.142 ± 0.233
Number of errors [median (min–max)]	0 (0–2)	1 (0–4)	0 (0–1)	1 (0–6)	0 (0–2)	0 (0–2)	0 (0–4)	0 (0–7)

Reaction times in seconds for correct trials (mean, SD) and number of errors [median (min–max)] for all conditions during the Emotional Face Assessment Task are presented.

As expected, Spearman’s rank correlation analyses showed strong intercorrelation between PIUQ subscales. Furthermore, positive correlations were found between PIUQ subscales and the scores of SABAS (Spearman’s rank correlation results are presented in Table 3).

**Table 3:** Correlational analyses of questionnaire data.

Questionnaire	PIUQ total (rho)	PIUQ obsession (rho)	PIUQ neglect (rho)	PIUQ control (rho)
PIUQ total	–	–	–	–
PIUQ obsession	0.798***	–	–	–
PIUQ neglect	0.872***	0.603***	–	–
PIUQ control disorder	0.741***	0.309*	0.594***	–
SABAS	0.520***	0.512***	0.468***	0.317**

Rho–Spearman’s correlation coefficient; *PIUQ* Problematic Internet Use Questionnaire; *SABAS* Smartphone Application-Based Addiction Scale; *P < 0.05, **P < 0.01, ***P < 0.001; Significant correlations after Benjamini–Hochberg correction are written in bold.

### Functional connectivity analysis results

For the left amygdala based functional connections, no association remained significant after Benjamini–Hochberg corrections (Table 4).

**Table 4:** ROI analysis results with left amygdala seed region.

ROIs of the FC	PIUQ total		PIUQ obsession		PIUQ neglect		PIUQ control		SABAS	
	rho	*P*-value	rho	*P*-value	rho	*P*-value	rho	*P*-value	rho	*P*-value
Right PCG	0.124	0.325	0.180	0.150	0.080	0,526	0.144	0.254	0.093	0.466
Left PCG	0.097	0.441	0.164	0.191	0.072	0.569	0.092	0.466	0.060	0.637
Right ACG	0.239	0.055	0.231	0.064	0.156	0.215	0.242	0.052	0.217	0.082
Left ACG	0.299	0.016	0,304	0.014	0.206	0.100	0.273	0.028	0.232	0.063
Right anterior SMG	0.100	0.430	0.209	0.095	0.011	0.928	0.052	0.682	0.115	0.360
Right posterior SMG	0.052	0.680	0.182	0.147	-0.061	0.628	0.058	0.644	0.117	0.354
Left anterior SMG	0.181	0.150	0.211	0.091	0.142	0.258	0.127	0.313	0.149	0.237
Left posterior SMG	0.108	0.390	0.181	0.148	0.029	0.820	0.109	0.387	0.152	0.228
Right anterior MTG	0.219	0.080	0.315	0.011	0.150	0.233	0.086	0.496	0.176	0.162
Right posterior MTG	0.126	0.316	0.161	0.199	0.038	0.765	0.152	0.227	0.072	0.568
Left anterior MTG	0.321	0.009	0.331	0.007	0.290	0.019	0.208	0.096	0.093	0.459
Left posterior MTG	0.222	0.075	0.218	0.081	0.134	0.287	0.205	0.102	0.012	0.927
Right SFG	0.127	0.313	0.130	0.301	0.080	0.524	0.108	0.391	0.158	0.208
Left SFG	0.224	0.073	0.146	0.245	0.232	0.063	0.185	0.140	0.190	0.130
Right FP	0.183	0.144	0.161	0.201	0.132	0.293	0.172	0.171	0.149	0.237
Left FP	0.252	0.043	0.203	0.104	0.250	0.045	0.240	0.054	0.167	0.183

Positive correlations between questionnaire scores and FCs. Rho- Spearman’s correlation coefficients and uncorrected *P*-values are presented; PIUQ–Problematic Internet Use Questionnaire; SABAS–Smartphone Application-Based Addiction Scale; PCG-posterior cingulate gyrus. ACG-anterior cingulate gyrus; SMG-supramarginal gyrus, MTG-middle temporal gyrus; SFG-superior frontal gyrus; FP-frontal pole.

After Benjamini–Hochberg correction, the scores of PIUQ obsession showed positive associations with the FCs between the right amygdala and the left PCG, bilateral ACG, right posterior SMG, bilateral posterior and anterior MTG and bilateral FP. Positive association between PIUQ neglect scores and the FCs between the right amygdala and left ACG and left FP remained significant after Benjamini–Hochberg correction. Only the positive association between PIUQ neglect scores and the FC between right amygdala and right posterior MTG remained significant after Benjamini–Hochberg correction. After corrections, PIUQ total scores correlated positively with the FCs between right amygdala and the bilateral ACG, bilateral PCG, right anterior and posterior MTG, left anterior MTG and bilateral FP (Table 5).

**Table 5:** ROI analysis results with right amygdala seed region.

ROIs of the FC	PIUQ total		PIUQ obsession		PIUQ neglect		PIUQ control		SABAS	
	rho	*P*-value	rho	*P*-value	rho	*P*-value	rho	*P*-value	rho	*P*-value
Right PCG	**0.298**	**0.016**	0.293	0.018	0.247	0.047	0.230	0.065	0.224	0.073
Left PCG	**0.317**	**0.010**	**0.324**	**0.009**	0.256	0.039	0.187	0.137	0.211	0.092
Right ACG	**0,382**	**0.002**	**0.357**	**0.004**	0.298	0.016	0.315	0.011	0.284	0.022
Left ACG	**0.374**	**0.002**	**0.330**	**0.007**	**0.325**	**0.008**	0.286	0.021	0.289	0.019
Right anterior SMG	0.257	0.038	0.230	0.065	0.254	0.041	0.141	0.261	0.219	0.079
Right posterior SMG	0.251	0.044	**0.289**	**0.020**	0.237	0.057	0.134	0.289	**0.305**	**0.014**
Left anterior SMG	0.116	0.357	0.133	0.290	0.113	0.371	0.029	0.013	0.444	0.001
Left posterior SMG	0.223	0.074	0.131	0.297	0.221	0.077	0.218	0.081	0.241	0.053
Right anterior MTG	**0.330**	**0.007**	**0.385**	**0.002**	0.223	0.074	0.221	0.076	**0.341**	**0.005**
Right posterior MTG	**0.348**	**0.004**	**0.324**	**0.009**	0.270	0.030	**0.305**	**0.014**	0.237	0.057
Left anterior MTG	**0.421**	**0.001**	**0.427**	**0.001**	0.332	0.007	0.252	0.043	0.236	0.059
Left posterior MTG	0.371	0.002	**0.342**	**0.005**	0.281	0.023	0.280	0.024	0.276	0.026
Right SFG	0.195	0.120	0.215	0.086	0.179	0.153	0.125	0.322	0.266	0.032
Left SFG	0.255	0.041	0.222	0.075	0.278	0.025	0.124	0.325	**0.315**	**0.011**
Right FP	**0.375**	**0.002**	**0.312**	**0.011**	0.301	0.015	0.313	0.011	**0.356**	**0.004**
Left FP	**0.467**	**0.001**	**0.420**	**0.001**	**0.373**	**0.002**	0.331	0.007	**0.485**	**0.001**

Positive correlations between questionnaire scores and FCs. Rho- Spearman’s correlation coefficients and uncorrected *P*-values are presented; *PIUQ* Problematic Internet Use Questionnaire; *SABAS* Smartphone Application-Based Addiction Scale; *PCG* posterior cingulate gyrus. *ACG* anterior cingulate gyrus; *SMG* supramarginal gyrus, *MTG* middle temporal gyrus; *SFG* superior frontal gyrus; *FP* frontal pole; Significant correlations after Benjamini–Hochberg correction (q = 0.05) are written in bold.

SABAS scores showed positive associations with the FCs between right amygdala and the right posterior SMG, right anterior MTG, left SFG and bilateral FP after Benjamini–Hochberg correction (Table 5).

## Discussion

In the present study we investigated the effect of problematic Internet use and excessive smartphone use (hereafter PIU&ESU) on the functional connections of the bilateral amygdala during the facial emotion recognition task. According to our results, the extent of PIU&ESU showed associations with amygdala’s functional connections in relation with cognitive control functions and social cognition.

The FCs between the right amygdala and frontal regions (frontal poles and superior frontal gyri) positively correlated with questionnaire scores (PIUQ and SABAS). The SFG is mainly involved in the organization of behaviour via cognitive control^55^. Related to this, the SFG is a core region in emotion regulation processes^56^, since via its’ orbitofrontal connection regulates the amygdala’s appraisal for emotional stimuli^23^. FP is a part of the prefrontal cortex (PFC)^57^ which plays a central role in organising behaviour via its top-down control function during emotion perception^16,58,59^. Fronto-striatal FCs, especially the connection between the dorsolateral PFC (dlPFC) and amygdala^60^ are essentials in emotion regulation and recognition^61,62^. The dlPFC, the ventrolateral PFC and the SFG are parts of the cognitive control network (CCN)^22,63,64^. The CCN is responsible for the monitoring and representing of contextual information, in service of organizing, controlling, and implementing human behaviour (such as emotion recognition) in harmony with the monitored environmental context^63,64^. Via cognitive control functions the CCN can regulate amygdala’s sensitivity towards emotional cues in according to the environmental context and behavioural goals^16,23,65^. Impairments within the CCN go hand in hand with disrupted emotion regulation, which contributes to the development and maintenance of addictive disorders^64^. Dysfunctional emotion regulation processes previously reported in Internet gaming disorder^66^, might also appear in PIU^67,68^ and ESU^69^. According to these findings, associations between questionnaire scores and the FCs in CCN are crucial in PIU and refer to the impairment of emotion regulation processes.

Our results are also related to social cognitive functions. The FCs between right amygdala and emotion recognition related brain regions (MTG, ACG, PCG and SMG) correlated positively with questionnaire scores (PIUQ, SABAS). SMG plays a key role in the EFE recognition via its’ somatosensory involvement. We understand someone’s emotional state by generating internal somatosensory images of it, to simulate within ourselves how others feel themselves^26^. Therefore, somatosensory regions, such as the anterior part of SMG, in functional cooperation with the amygdala are indispensable in the recognition of socially relevant information (such as emotion) from faces^26,70^. The MTG is involved in language processes^71^, but also related to emotion perception and social cognitive processes such as mentalizing functions^18,72,73^. Since EFEs provide necessary information about others’ mental state^14,15^, mentalizing is indispensable in appropriate EFE recognition. The PCG and ACG in functional cooperation with the amygdala contribute to emotional face expression recognition^17^ and social cognitive evaluation processes^74^. The PCG is involved in mentalizing^75^ and in the regulation of attentional processes toward relevant social cues^76^. The ACG is related to action monitoring in self and others^77^ and emotional evaluative and regulative mechanisms^78,79^. Via its functional connection with the dlPFC, ACG also takes part in the attentional control of emotional relevant context^17^. Alterations within resting-state FCs between ACG and amygdala were previously found in PIU^34^. Moreover, impaired task-based FCs between these regions were found in cocaine^30^ and methamphetamine^31^ users during emotion recognition, which were associated with socially maladaptive behaviours^31^. Similarly, poorer performance in emotion recognition was previously reported among SNS addicts related to controls^32^. Furthermore, the performance of emotion recognition in IGD subjects is an independent predictor of the extent of the addiction^33^. Based on our own and the above cited findings, social cognitive functions, such as mentalisation, but especially the emotional face expression recognition are altered in PIU&ESU.

There are some limitations which must be considered. First, the scores of PIUQ and SABAS showed no correlations with the behavioural performance (RTs, ERs). The most plausible explanation is that the FERP was relatively easy to complete and was not sensitive enough to detect individual differences. It must be noted that the essence of the task was not to detect individual differences rather to get proper amygdala seed time-courses and its’ functional connections for PPI, since the task effectively activates emotion recognition-related cortico-limbic networks, including the amygdala^44^. Secondly, only five correlations between FCs and PIUQ scores remained significant after Benjamini-Hochberg corrections (Table 4 and Table 5). Therefore, some of our results should be interpreted as less robust evidence and further studies are needed to verify them. Finally, the cross-sectional nature of our investigation limits the possibility of interpretation of what are causes and effects in the relationship between social cognition and PIU&ESU. We do not know if PIU&ESU leads to social cognitive difficulties or vice versa. To understand the nature of the relationship, longitudinal studies are highly needed.

In conclusion, our results released two different functional brain systems that are altered in PIU&ESU. First, the involvement of CCN was revealed. Altered functioning of fronto-striatal FCs are essential in the development and maintenance of addictive disorders^64^. Decline in cognitive control functions is a common phenomenon among different addictions induced by the continuous compensation of addictive behaviour^80,81^. Second, the involvement of social cognitive functions (mentalisation and EFE recognition) in PIU&ESU was revealed. The FCs between the amygdala and ACG, PCG, MTG and SMG are essential in intact social cognitive functioning^17,18,26^. Altered functioning of this connections have been previously described in different substance use disorders^27–31^ which connected to poorer performance in emotion recognition and socially maladaptive behaviours. These findings help to understand a slice of the altered social cognitive functioning in PIU&ESU and give a well-established starting point for further investigations. During the past decade Internet technology changed our daily life and the way we communicate as well. A significant proportion of social interactions take place in online space*,* individuals keep in touch more easily via the Internet and time spent with others maybe decreases in the real world. The long-term impact of this extensive social life and communication in online space is not well understood. Therefore, further investigation of social functioning is essential in PIU&ESU. To the best of our knowledge this is the very first study in which social cognitive functions and their neural correlates were investigated in PIU&ESU. Nevertheless, our results emphasized that besides the impairment in brain’s executive and reward systems, the social brain network might be the next candidate to be involved in the pathogenesis of PIU&ESU.

## Data Availability

The datasets generated during and/or analysed during the current study are available from the corresponding author on reasonable request.
